# The Common Task

**Published:** 1907-06-01

**Authors:** 


### THE COMMON TASK.

**Correspondence and Queries** for this section should be sent to the Editor of The Hospital, 28 Southampton Street, Strand, London, and marked "Nursing Administration."

### The Price of Beef Tea.

In compliance with the desire expressed by the managers of certain institutions to compare the price of ordinary beef tea with that of preparations made by the addition of boiling water to concentrated extracts, we have procured samples of nine different varieties of the latter, and now place before our readers in alphabetical order particulars of the cost per pint of making each kind, together with a general summary of the qualities and food value of each brew, so far as these have been communicated to us by the manufacturers.

*Invalid Bovril.*—This preparation is specially prepared for institution use, and is supplied packed in 7-lb. tins at the rate of 4s. 6d. net per lb. The quantity required for making one pint of beef tea is ⅝ oz., and the cost, therefore, works out at almost exactly 2¼d. per pint. The analysis of ordinary Bovril given in "Allen's Commercial Organic Analyses" is as follows:—

| | |
|---|---|
| Water ... ... ... ... ... | 21.16 |
| Insoluble proteids, meat fibre, etc. ... | 8.47 |
| Soluble proteids and gelatine ... ... | 8.19 |
| Meat bases ... ... ... ... | 16.13 |
| Non-nitrogenous extractive matter ... | 29.23 |
| Mineral matters ... ... ... ... | 16.82 |

It is claimed for the "Invalid Bovril," in addition, that it is specially enriched with soluble proteid, and provides a percentage of nitrogenous matter, "in accordance with the pronounced views of many dietetic authorities."

*Brand's Fibrous Beef Tea.*—"This is made from the finest fresh British beef." Sold in three-lb. tins, for the supply of hospitals and other charitable institutions at 10d. per lb. The three-lb. tin is estimated to produce at least seven pints of strong beef tea. The cost works out at 4¼d. per pint.

*Lemco and Oxo, and Nursing Oxo.*—Liebig's Extract of Meat Co. Lemco, formerly known as Liebig's Extract, packed in 7-lb. jars for institution use, is sold at 5s. 6d. per lb. One pound is estimated to produce 60 pints, the cost, therefore, working out, when bought in large quantities, at 1 1-10d. per pint. It is "concentrated essence of beef without any addition whatever; it contains no fat."

*Oxo.*—Sold in 7-lb. tins for institution use at 3s. 9d. per lb. It is estimated that 22 pints can be made to the pound. The cost is, therefore, 2d. per pint.

*Nursing Oxo.*—This is a peptonised and unflavoured preparation of Oxo. It is claimed that the inclusion of meat peptone gives it additional value as a liquid food in illness. Sold in 14-lb. tins it costs 4s. per lb., and at 22 pints to the pound it works out at a cost of 2 1-5d. per pint.

*Ramornie.*—Liebig's Extract of Meat, as prepared by the Australian Meat Company. Supplied in large quantities for institution use at about 5s. per lb. The amount given in the directions for making beef tea is ¼ oz. to ¾ pint of water. The cost, therefore, works out at a little over a penny a pint.

*Mason's Beef Tea.*—George Mason and Co. "Contains not only the extractives of the meat, but the muscular fibre also in a finely comminuted state." According to the *Lancet* analysis, "the amount of meat fibre is 3.74 per cent. The extractives and albuminous matters in solution amount to 12.95 per cent., of which 2.37 per cent. is due to mineral salts." The cost of this preparation, as supplied in bulk to hospitals, works out at rather less than 3½d. a pint.

*Oxvil.*—F. L. Borthwick and Co. Two ounces of this preparation will make one pint of beef tea, and the cost works out at 5d. per pint. "Contains the albumen and fibrin, together with the soluble extract of fresh beef."

Lastly we have ordinary beef tea, which may be prepared in various ways and at a widely different cost. The usual method we have found practised in institutions is to shred the meat as fine as possible, to add cold water in the proportion of a pint to a pound, and to stew gently for several hours. The cost varies, as will be seen with the price of the beef. The average is 4d. a pound, though this is considerably more than need be paid. Dr. Rideal's experiments went to prove that in making beef tea after this fashion a large residue of nutritious matter remained adhering to the rags of beef after the infusion had been strained off; if a second infusion were made the cost of the tea was reduced in proportion to the amount of water subsequently added. Moreover, foreign meat can be obtained at prices varying according to the state of the market, from 2d. to 3d. a pound, or even less when the whole shin is purchased. Thus, it would be possible to reduce the price of ordinary beef tea to 1½d. or 2d. a pint. No analysis can be given of ordinary beef-tea for the reason that it differs enormously in results even when carefully prepared from the best recipes. It has been calculated that for every 100 beds a hospital consumes something like 10,000 pints of beef tea in a year. The question, therefore, whether to use prepared beef tea, or that made on the premises from fresh beef, is seen to be one of considerable importance. We propose to consider the matter from a different standpoint on another occasion.

### WASTE IN POOR-LAW INFIRMARIES

We are interested to see that the question of avoiding waste in these institutions has been engaging the attention of the Society of Poor-law Workers, who had the problems connected with it under discussion at their meeting this week at Aubrey House, Kensington. It seems that the great bar to economical administration is the complicated system of accounts in use, and that until the accounts are simplified little can be done. We commend to the guardians a study of the "Uniform System of Accounts" (Scientific Press).

